# Hydrogen–water enhances 5-fluorouracil-induced inhibition of colon cancer

**DOI:** 10.7717/peerj.859

**Published:** 2015-04-07

**Authors:** Joshua Runtuwene, Haruka Amitani, Marie Amitani, Akihiro Asakawa, Kai-Chun Cheng, Akio Inui

**Affiliations:** 1 Department of Psychosomatic Internal Medicine, Kagoshima University Graduate School of Medical and Dental Sciences, Kagoshima, Japan; 2 Faculty of Medicine, Sam Ratulangi University, Manado, Indonesia

**Keywords:** Antioxidant, Hydrogen, Cancer, Apoptosis, 5-fluorouracil

## Abstract

Oxidative stress is involved in cancer development. Hydrogen (H_2_) is a potent antioxidant and exhibits anti-inflammatory and potentially anticancer-like activities. This study aimed to investigate the role of H_2_ incombination with 5-fluorouracil (5-FU) in cancer treatment both in vitro and in vivo using the colon 26 cell line. The survival rate was determined using the Kaplan–Meier survival test, and cell viability was assessed using cell viability imaging kit and the MTT assay, and activation of the cell apoptosis pathway (Phosphorylated adenosine monophosphate activated protein kinase (p-AMPK), Apoptosis-inducing factor (AIF) and Caspase 3) were characterized by western blots. Hydrogen water administration improved the survival of mice with colon 26-induced cancer. Furthermore, hydrogen water enhanced cell apoptosis in cancer cells, resulting in a marked increase in the expression of p-AMPK, AIF and Caspase 3 in colon 26 cells. Hydrogen water also increased the inhibitory effect of 5-FU on colon 26 cells with spect to cell survival rate and anticancer functions. Additionally, high-content hydrogen water exhibited stronger antioxidative and anticancer activity than did the natural hydrogen water. In conclusion, high-content hydrogen water can inhibit colon cancer, particularly in combination with 5-fluorouracil.

## Introduction

Hydrogen (H_2_) is an antioxidant agent able to scavenge reactive oxygen species (ROS) (Ohta, 2012). Specifically, hydrogen reduces hydroxyl radical (**⋅** OH, the most cytotoxic ROS) and peroxynitrite (ONOO^−^). Long-term exposure to ROS as oxidative stress may lead to the development of metabolic disorders and cancers (Casares et al., 2012; Meng & Zhao, 2012). Thus, hydrogen has been suggested as a suitable candidate for, or contributor to the therapeutic strategies for various diseases, including cardiovascular disease, cerebrovascular disease, metabolic disease (Aoki et al., 2012; Ghanizadeh & Berk, 2013; Iio et al., 2013; Nagatani et al., 2013; Nakao et al., 2010), respiratory disorders and certain types of cancer (Kawai et al., 2012; Ning et al., 2013). Hydrogen has emerged as a promising cancer remedy as a preventative agent or in combined therapy with anticancer drugs. Hydrogen water consumption might mitigate the side effects of anticancer drugs by decreasing oxidative stress and ameliorating metamorphosis due to decreased apoptosis (Nakashima-Kamimura et al., 2009). Hydrogen also exhibits radioprotective action by protecting the immune system (Yang et al., 2012). Furthermore, hydrogen may alleviate the hematological injury induced by radiation through the suppression of radiation-induced caspase 3 activation, in addition to rescuing the radiation-induced depletion of white blood cells (WBC) and platelets (Yang et al., 2012). Furthermore, hydrogen is not known to diminish the activity of anticancer drugs or radiation against cancer cells either *in vitro* or *in vivo* (Kang et al., 2011; Nakashima-Kamimura et al., 2009). Although anticancer properties of hydrogen have been suggested, the mechanism(s) and efficiency by which hydrogen inhibits cancer cells remained to be established.

5-fluorouracil (5-FU) is the first-line chemotherapeutic drug in the treatment of colon cancer due to its impact on the colorectal area and its ability to inhibit the growth of cancer cells by incorporating its metabolites into DNA and RNA (Longley, Harkin & Johnston, 2003). This compound is reported to be effective for the treatment of solid tumors and the prevention of liver metastasis of colon 26 adenocarcinoma cells in mice, and the combination of 5-FU with certain modalities of cancer therapy, including radiation and other anticancer drugs, enhances its anticancer effects (Ishikawa et al., 1989; Tominaga et al., 1993). Increased hydrogen bonding of fluorouracil has been proposed to enhance its function (Kugimiya, Mukawa & Takeuchi, 2001). In an attempt to understand the role of hydrogen rich-water in cancer treatment, we investigated the survival rate, cell apoptosis, and anticancer parameters of an animal model of cancer cachexia (colon 26-induced cancer mice) (Tanaka et al., 1990) treated with high-content hydrogen water (HHW) or natural hydrogen water (NHW) and 5-FU.

## Materials and Methods

### Cell line

The mouse colon carcinoma cell line (Colon 26) was purchased from the American Type Culture Collection (ATCC) (Manassas, Virginia, USA) through Summit Pharmaceuticals Intl. Corp. (Tokyo, Japan). Cells were cultured in ATCC-formulated RPMI-1640 medium supplemented with fetal bovine serum to a final concentration of 10%. The cells were maintained at 37 °C in a humidified 5% CO_2_ environment until approximately 60% confluence. The medium was then replaced with serum-free cell medium containing assorted 5-FU, HHW, NHW, 5-FU + HHW, and 5-FU + NHW treatment (Kyowa Hakko Kogyo Co. Ltd, Tokyo, Japan). After incubation for 24 h, the cells were harvested for further studies.

### Animals

Six-week old female BALB/c mice, weighing 19 to 24 g, were maintained in a pathogen-free environment under a 12 h light/12 h dark cycle with a controlled room temperature at the animal center of Kagoshima University (Kagoshima, Japan). Food and tap water were provided *ad libitum*. To develop cancers, 5 × 10^5^ viable Colon 26 cells in 100 µl RPMI-1640 medium were injected into the abdominal cavity by intraperitoneal injection (Perboni et al., 2008). All procedures in this study were approved by the Ethics Committee for Animal Care and Use of Kagoshima University (IRB approval number MD13062) and conducted by following the Japanese national standardized guideline for animal experiments of Kagoshima University.

### High-content hydrogen water

High-content hydrogen water was prepared in our laboratory by dissolving H_2_ in pure water under high pressure (0.4 MPa) for 24 h to achieve overall 0.8 mM hydrogen saturation. For comparison, natural H_2_ water (NHW) drawn from Mount Fuji (Yamanashi, Japan) containing 0.125 mM of hydrogen (VanaH Co., Ltd., Yamanashi, Japan) was also used in this study.

### Measurement of hydrogen concentration

#### Hydrogen waters and cell lines

We measured the hydrogen concentrations to confirm the adequate hydrogen concentration that is able to produce an antioxidant effect. Each hydrogen waters (HHW and NHW) was placed in a closed glass vessel (500 ml) and cell line dishes containing ATCC-formulated RPMI-1640 medium supplemented with fetal bovine serum to a final concentration of 10%. Hydrogen concentrations were then measured by hydrogen electrode (DK-8200 Aarhus N; Unisense, Denmark) as previously described with some modifications (Amitani et al., 2013).

### In animals

The animals were seperated into 5 groups (5-FU, HHW, NHW, 5-FU + HHW, and 5-FU + NHW). Hydrogen waters 0.6 ml (HHW and NHW) was force-fed into the mice’s stomach using gavage. At each predetermined timepoint (0, 2, 4, 6, 8 h), 150 µl of venous blood was withdrawn, and the hydrogen concentration was measured by hydrogen electrode.

### Cell viability assay

The Live/Dead Cell Imaging Kit (Life Technologies, Carlsbad, California, USA) was qualitatively used to examine cell viability. Live green (1 ml of 1 µM) solution was dissolved into dead red solution (1 mM lyophilized stock). Colon 26 cells and HepG2 cells were treated with a 2× equal volume of testing solution for 1 h. After incubation for 15 min at room temperature, cell images were captured by microscopy.

Cell viability was also determined using the 3-(4,5-dimethylthiazol-2-yl)-2,5-diphenyltetrazolium bromide (MTT) assay. Five treatments (5-FU, HHW, NHW, 5-FU and HHW, and 5-FU and NHW) were applied to 10^4^ colon 26 cells in 96-well plates for 24 h. The medium was then substituted with 200 µl/well free medium in each well (5-FU group final dose: 50 µM). After incubation for 4 h at 37 °C, dark blue formazan crystals were formed by the viable cells, and 0.01 M HCl/10% SDS was added. Synergy HT Multi-mode Microplate Reader (Bio-Rad, Hercules, California, USA) was then used at 570 and 600 nm to quantify the absorbance of each well as described previously (Yu et al., 2012). The data were collected from triplicate experiments.

### *In vivo* and *in vitro* experiments

*In vivo*: after the confirmation of tumor induction in all groups except the cancer-free mice drinking hydrogen water, mice were treated every 8 h with MQ water (Milli-*Q* Integral per produce), HHW or NHW by oral administration (250 µl), and 5-FU dissolved in a 5% glucose solution was administered (100 mg/kg) by intraperitoneal injection weekly (Perboni et al., 2008). At ten days after tumor induction, the mice were sacrificed, and the tumors were excised and scaled. Tumor size is measured with caliper and calculated using the following equation: Length × width^2^ × 0.5, as previously described (Lee et al., 2012).

*In vitro* A solution of 50 µl MQ, HHW (0.8 mM) or NHW (0.125 mM) was added to each slide chamber (IWAKI, Japan), and the results were compared with slides chambers administered only 5-FU at a final dose of 50 µM.

### Western blot analysis

The expression of p-AMPK, AIF, and Caspase 3 was examined using western blotting analysis. Ice-cold radio-immuno-precipitation assay (RIPA) buffer supplemented with phosphatase and protease inhibitors (50 mM sodium vanadate, 0.5 mM phenylmethylsulphonyl fluoride, 2 mg/ml aprotinin, and 0.5 mg/ml leupeptin) was used for protein extraction and elimination of homogenates and cell lysate. The protein concentrations were then measured by BCA protein assay (Thermo Fisher Scientific Inc., Waltham, Massachusetts, USA). Total protein samples (30 µg) were filtered via SDS-PAGE (Polyacrylamide Gel Electrophoresis) (10% acrylamide gel) using the Bio-Rad Trans-Blot system and were transferred to membranes. The membranes were blocked with 5% non-fat milk in Tris-buffered saline containing 0.1% Tween 20 (TBS-T), incubated for one hour, washed in TBS-T and hybridized with primary antibodies diluted to a suitable concentration in TBS for 16 h. Afterwards, specific antibodies for AMPK and p-AMPK (Cell Signaling, Danvers, Massachusetts, USA), AIF (Merck Millipore, Billerica, Massachusetts, USA), and Caspase 3 (Merck Millipore, Billerica, Massachusetts, USA) were used. Additionally, membranes were incubated with secondary antibody to bind the *β*-actin (Merck Millipore, Billerica, Massachusetts, USA) (1:5,000 dilution), which served as the internal control. Incubation with secondary antibodies and the detection of the antigen-antibody complex was performed using an ECL kit (Amersham Biosciences, Amersham, UK). After comparing with the marker for specificity, the immunoblots of p-AMPK (62 kDa), AMPK (62 kDa), *β*-actin (43 kDa), AIF (57 kDa), and Caspase 3 (17 kDa) were quantified with a laser densitometer.

### Free radical scavenging assay

The free radical scavenging activity of H_2_ was determined using a 2,2-diphenyl-1-picryl-hydrazyl-hydrate (DPPH) radical scavenging photometric assay and compared to the activity of vitamin C. Twenty microliters of each sample solution [0.01-1 mM of vitamin C (Sigma-Aldrich, Seelze, Germany), 0.01–0.8 mM of H_2_ (HHW), and 0.0001–0.1 mM of H_2_ (NHW)] and 200 ml of DPPH solution [2 mM of 2, 2 diphenyl-1-picryl-hydrazyl (Sigma-Aldrich, Seelze, Germany) prepared in methanol] were added to each micro plate. Samples were then incubated in the dark at room temperature for 10 min, and the absorbance (Ab) was measured at 540 nm using a microplate reader. Radical scavenging activity was calculated using the following equation: % antioxidant activity = [12(Ab of sample/Ab of blank)] ×100, as described previously (Amitani et al., 2013).

### Statistical analysis

The data are presented as the means ±SEM with respect to the number of samples (*n*) in each group. Statistical analysis wa s conducted using repeated measures one-way analysis of variance and two-way analysis of variance (ANOVA). Tukey’s test was used to determine significant differences. Results with *p* < 0.05 were considered statistically significant. The survival rates were analyzed using the Kaplan–Meier survival test and the statistical analysis is performed using log rank test with *p* < 0.05 being considered as significant.

## Results

### Hydrogen water increases the survival rate of colon 26-bearing mice

**Figure 1:**
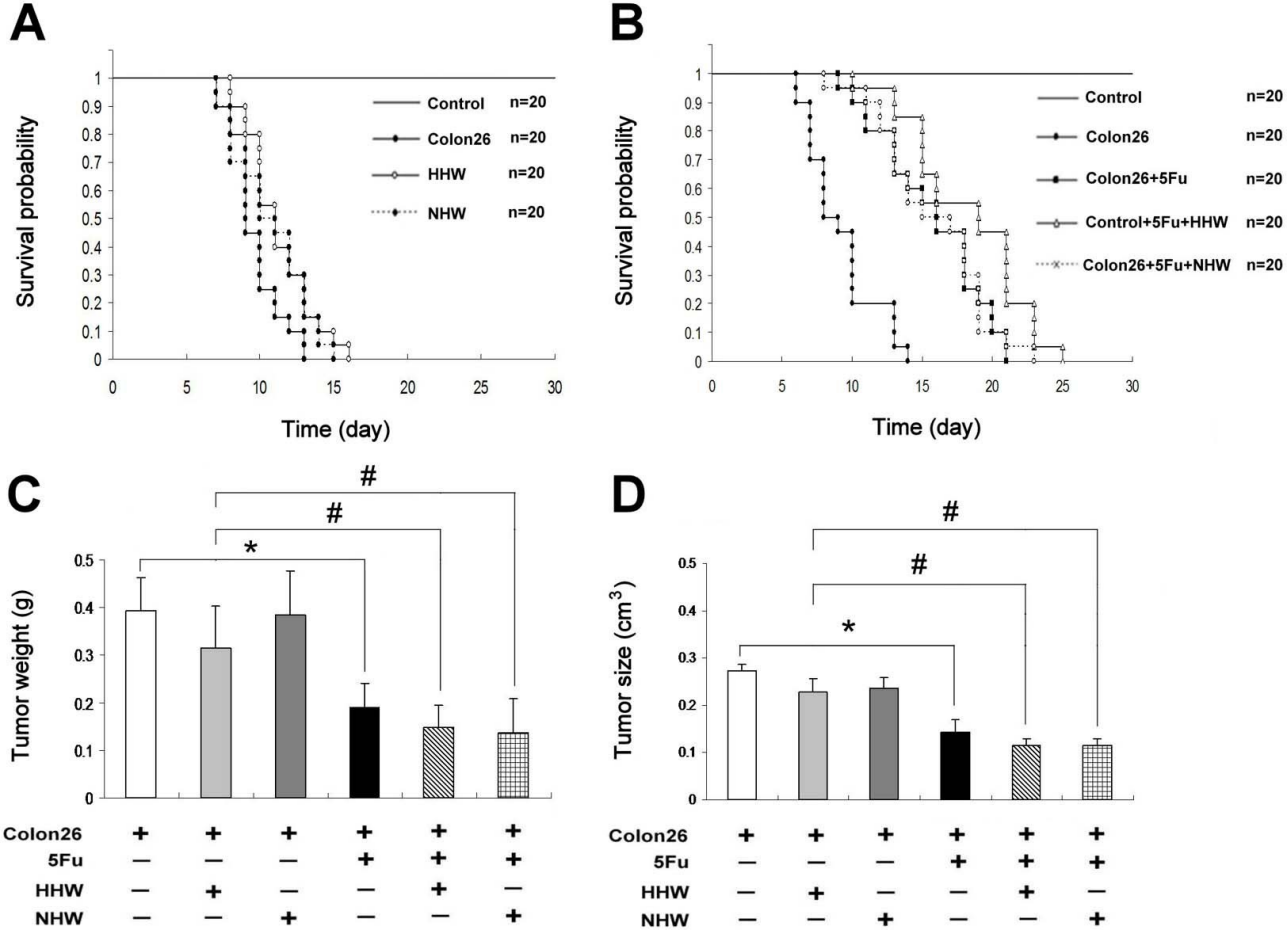
Kaplan–Meier. The survival rate of the HHW-treated group was slightly higher than the NHW group. The addition of 5-FU on day 14 significantly prolonged the survival rates (*P* < 0.05) of colon 26-bearing mice. Combination therapy of HHW and 5-FU produced the longest survival rate, followed by the combination NHW with 5-FU and 5-FU therapy alone. The result of log rank test analysis: for Colon26 and HHW, *P* < 0.05 (Fig. 1A); colon26 and Colon26 + 5FU, *P* < 0.001; Colon26 and Colon26 + 5FU + HHW, *P* < 0.001; Colon26 and Colon26 + 5FU + NHW, *P* < 0.001; and Colon26 + 5FU and Colon26 + 5FU + HHW, *P* < 0.05. The tumor size and weight are shown in Fig. 1C and 1D. The 5-FU group was found to be significantly decreased compared to the control group in both tumor weight and size (*n* = 7, ^∗^*p* < 0.05). Similarly the tumor weight and size was further decreased in H_2_ treated groups of 5FU + HHW and 5FU + NHW (*n* = 7, ^#^*p* < 0.05).

The survival rate in the groups treated with only H_2_ water (HHW and NHW) was slightly increased relative to the group of cancer-bearing mice that did not receive treatment (Fig. 1A). Moreover, the survival rate in the HHW-treated group was significantly greater than that in the NHW group (Fig. 1A). After treatment with 5-FU, the survival rate significantly increased (Fig. 1B). The combination of HHW with 5-FU exhibited a marked increase in survival rate (*p* < 0.05) , whereas the combination of NHW with 5-FU was less effective (Fig. 1B). Water intake was monitored throughout the experimental period and was nearly the same among the groups. Body weight and food intake were also monitored and were significantly lower in the colon 26 tumor-bearing mice group than in the control group (data not shown). However, no significant difference was observed in body mass recovery between the hydrogen water treated group and the control group (data not shown). The tumor size and weight of the 5-FU group is significantly decreased compared to the control group (Figs. 1C and 1D).

### Hydrogen attenuation rate *in vitro* and *in vivo*

**Figure 2:**
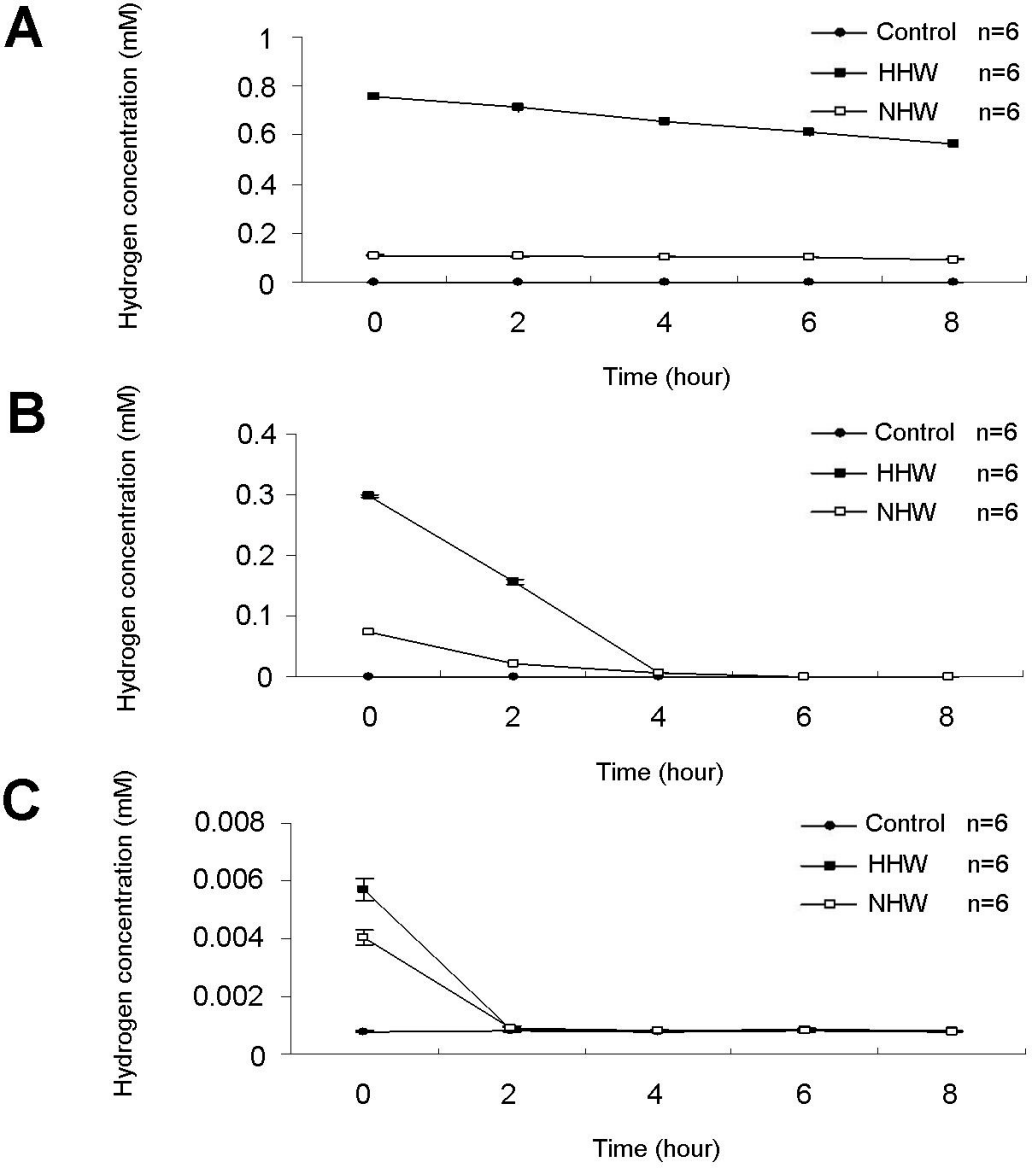
The hydrogen (H_2_) concentration of three treatments (HHW, NHW, and control) over an 8-h period. (A) Direct examination of H_2_ concentrations in each container, (B) H_2_ concentrations in the in vitro experiment, and (C) H_2_ concentrations in the in vivo experiment as measured in the blood after direct catheterization of hydrogen water into the animal’s stomach.

Hydrogen concentrations were monitored for up to 8 h (Figs. 2A–2C). Fresh preparations of HHW exhibited maximum H_2_ concentrations of nearly 0.8 mM, whereas NHW maintained an H_2_ concentration of 0.1 mM (Fig. 2A). In cell culture media, the H_2_ concentration of HHW reached a maximum concentration of 0.3 mM, whereas NHW reached a maximum concentration of approximately 70 µM (Fig. 2B). In mice, the hydrogen concentration in the blood peaked at 6 µM for HHW and 4 µM for NHW (Fig. 2C).

### Hydrogen water enhances the cancer cell apoptotic effect of 5-FU

**Figure 3:**
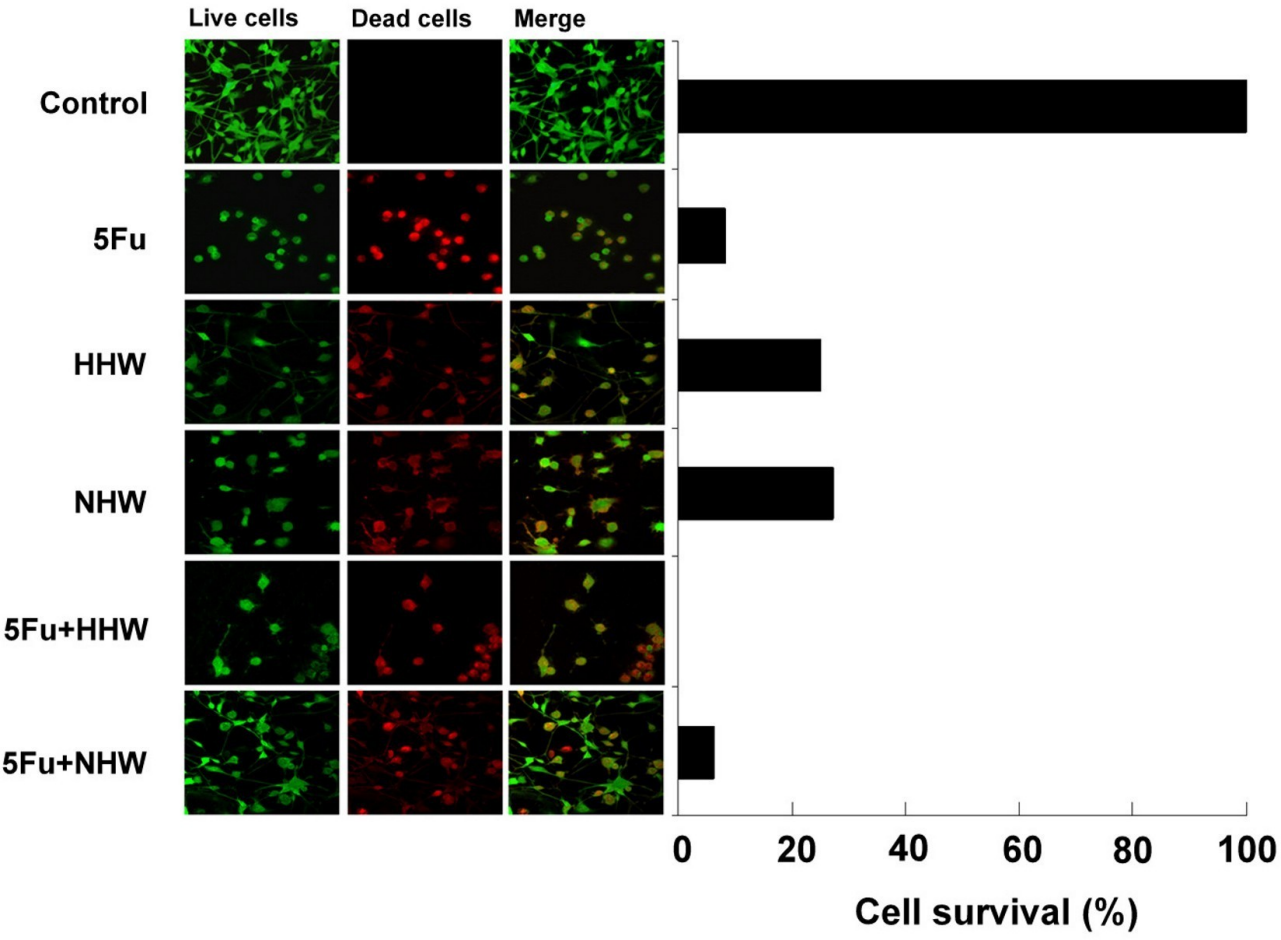
The cell viability of colon 26 cells 4 h after treatment with 5-FU, HHW, NHW, 5-FU+ HHW, and 5-FU+ NHW. The administration of 5-FU decreased the viability of colon 26 cells; H_2_ water (HHW and NHW) also decreased viability but to a lesser extent. Combined treatment of 5-FU+ HHW exhibited the greatest cell apoptosis in colon 26 cells, followed by a combination of 5-FU and NHW.

The cell viability of colon 26 cells was assessed after a 24-h incubation with 5-FU, hydrogen water (HHW, NHW) or a combination of 5-FU with hydrogen water. As shown in Fig. 3, the combination of 5-FU and HHW produced the greatest induction of cell apoptosis in colon 26 cells, followed by the combination of 5-FU and NHW. The administration of H_2_ water (HHW and NHW) resulted in a decrease in viability of colon 26 cells, but the effectiveness was lower than the treatment of 5-FU.

**Figure 4:**
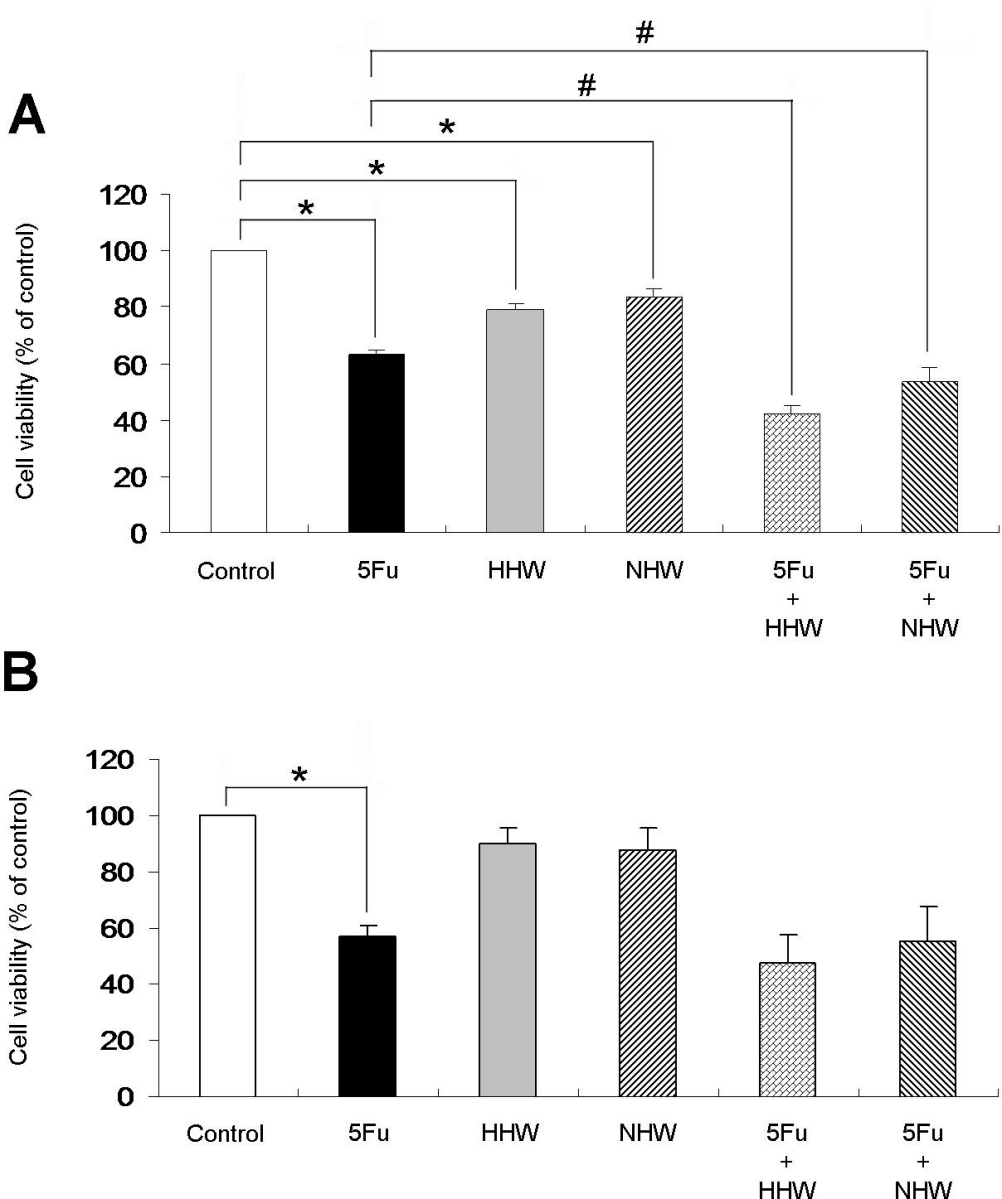
Cell viability of colon 26 (A) and HepG2 (B) were measured by MTT assay. The cell viability of colon 26 cells was significantly reduced (*p* < 0.05) in all of the treatment groups (5-FU, HHW, NHW) compared with the control group. 5-FU decreased the cell viability of colon 26 cells most significantly, followed by HHW and NHW, respectively. Combined therapy of 5-FU and HHW or NHW decreased cell viability (*p* < 0.05) relative to HHW alone. 5-FU but not H_2_ decreased cell viability of HepG2.

The cell viability of colon 26 cells was then examined by MTT assay (Fig. 4A). All treatments by each agent (5-FU, HHW, NHW) in this experiment were effective in reducing the cell viability of colon 26 cells compared with the control. In isolation, treatment with 5-FU was most effective, followed by HHW and NHW. In combination, HHW with 5-FU resulted in marked cell apoptosis in colon 26 cells, and NHW with 5-FU water produced a similar result. However, H_2_ did not show any statistical significant effects in HepG2 cells (Fig. 4B)

### HHW exhibits a stronger anti-oxidative effect than NHW

**Figure 5:**
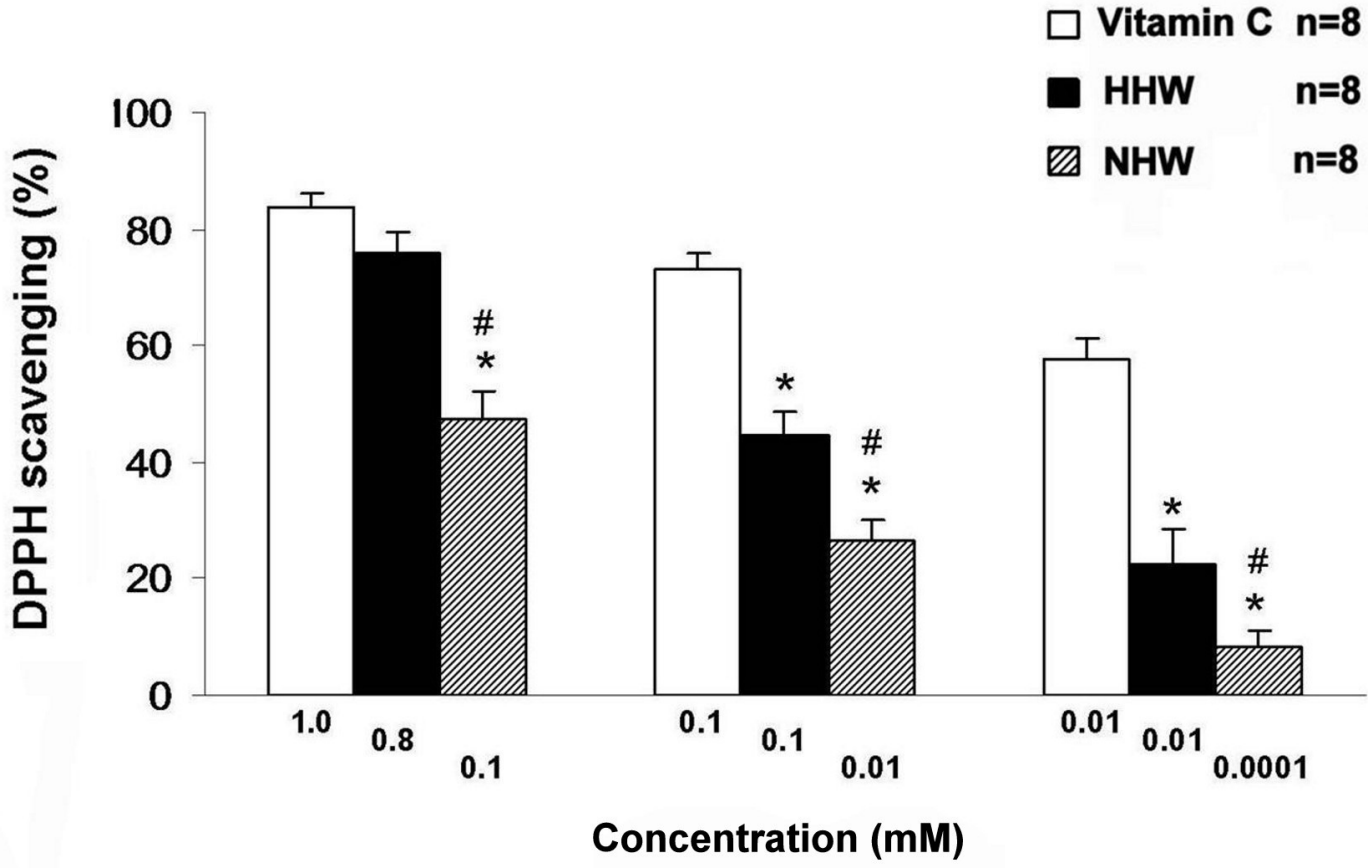
The free-radical (DPPH) scavenging activity of HHW and NHW in comparison with Vitamin C at several ranges of the doses. ^∗^*p* < 0.05 for the difference between Vitamin C and HHW. ^#^*p* < 0.05 for the difference between Vitamin C and NHW.

The anti-oxidative assay, which measures free radical (DPPH) scavenging activity, indicated that both HHW (0.01 and 0.8 mM) and NHW (0.01 and 0.1 mM) exhibited antioxidant activity (Fig. 5). Additionally, HHW exhibited a stronger anti-oxidative effect than NHW, approaching the anti-oxidative action of Vitamin C (1 mM). At each maximum dose Vitamin C (1.0 mM) has highest anti-oxidative effect followed by HHW (0.8 mM ) and NHW (0.1 mM ) respectively. However, at mole/mole comparison, HHW (0.1 mM ) and NHW (0.1 mM ) have the same level of anti-oxidative effect.

### Identification of cell apoptosis in colon 26 cells via p-AMPK, AIF and Caspase 3 expression

**Figure 6:**
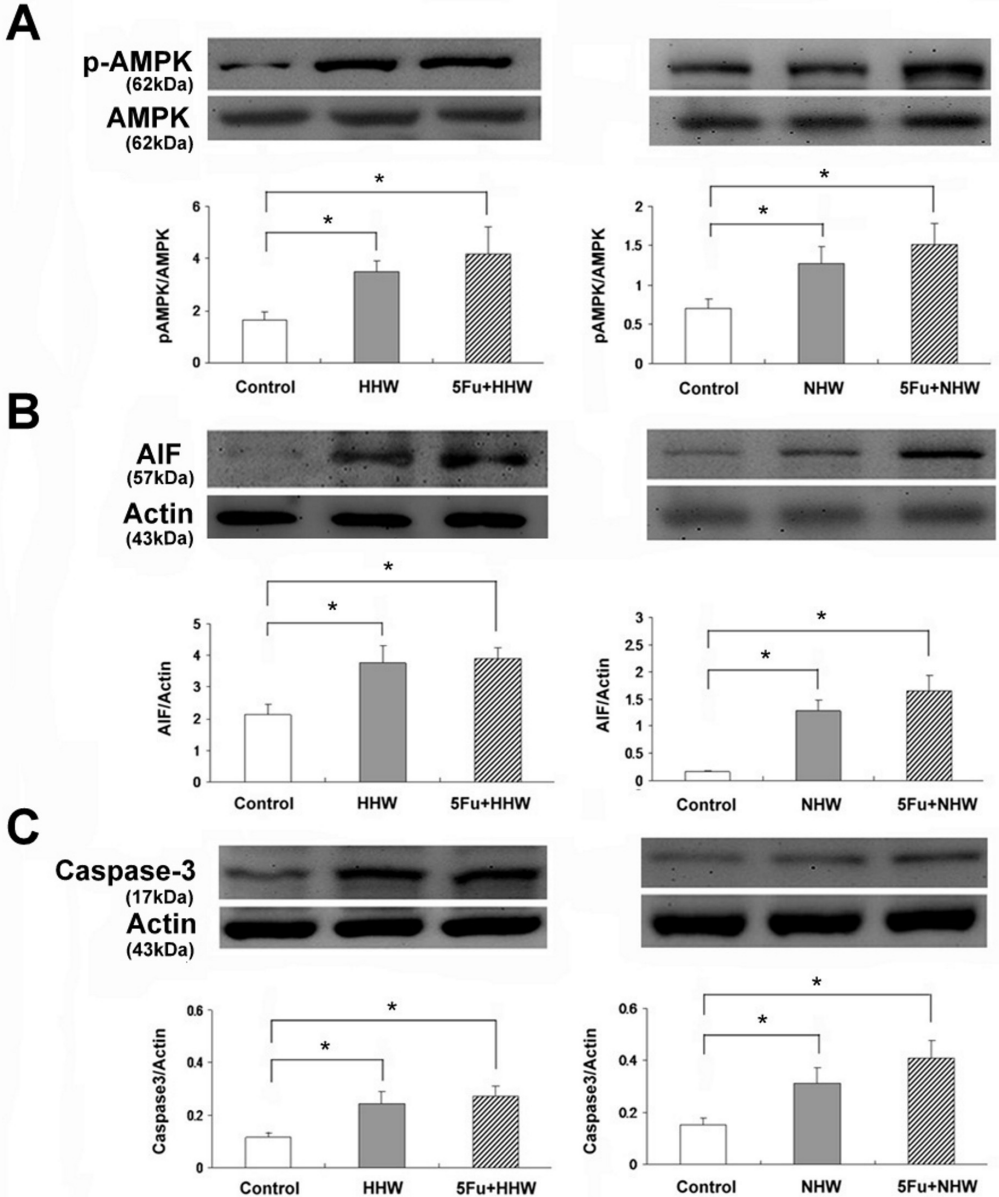
Western blot analyses of p-AMPK, AIF and Caspase 3 for cell apoptosis in colon 26 cells after treatment with HHW or NHW and in combination with 5-FU. The expression of p-AMPK, AIF and Caspase 3 were significantly increased after administration of both H_2_ water treatments (HHW and NHW). The levels of all examined proteins were increased by the addition of 5-FU. Higher expression was observed after treatment with HHW than after treated with NHW.

The expression of p-AMPK, AIF and Caspase 3 in colon 26 cells was elevated after treatment with both hydrogen waters (HHW and NHW). The expressions of these proteins were further increased in colon 26 cells after the combined treatment with 5-FU, as shown in Fig. 6.

## Discussion

Conventionally, hydrogen administration by inhalation requires a professional technician and sophisticated equipment (Huang et al., 2010; Zhang et al., 2012). However, hydrogen administration through water is simpler and does not require any special equipment (Zhang et al., 2012), and its efficacy and impact are similar to inhalation (Shimouchi et al., 2013). Hydrogen water is also preferable due to its simple production and the fact that it can be administered safely to the human body. Therefore, hydrogen water is more convenient and applicable than conventional inhalation administration, particularly in preventive care. This therapy is also simple for out-patients. In this study, we adopted the popular method to prepare H_2_ water using the high-pressure vacuum method as described previously (Karthikkumar et al., 2012; Zeng et al., 2014; Zhang et al., 2012). Other methods, including a metallic magnesium stick and alkaline water electrolysis (Nakao et al., 2010; Qin et al., 2012; Zeng et al., 2014), are also able to generate adequate hydrogen saturation. Thus, many methods can be applied according to individual preference and availability. The major challenge in the production of hydrogen water is preservation of hydrogen ions contained in the dilution, as the number of hydrogen ions is prone to decrease (Kajiyama et al., 2008). In contrast to several methods that have been developed to overcome this issue (Kang et al., 2011; Nakao et al., 2010; Ohta, 2011), we decided to provide and replace H_2_ water daily in this study. In accordance with a previous report (Nagata et al., 2009), the administration of hydrogen 0.8 mM (HHW) via the stomach resulted in blood concentrations of hydrogen of approximately 6 µM, whereas the administration of 0.1 mM (NHW) surprisingly resulted in peak blood hydrogen levels of 4 µM. The similarity in these values may be the result of the supplementation of salts such as sodium chloride, which are believed to contribute to maintaining H_2_ saturation in water (Amitani et al., 2013). Thus, both HHW and NHW resulted in adequate concentrations of H_2_*in vivo*.

As shown in the survival curves, treatment with H_2_ water alone did not prolong the survival rates in cancer-bearing mice, although a trend toward improved survival was observed (Fig. 1). 5-FU was applied in this study because it is an antimetabolite drug used clinically for treatment of colorectal cancer (Mhaidat, Bouklihacene & Thorne, 2014). 5-FU produces anticancer effects via inhibition of thymidylate synthase (TS) and the integration of its metabolites into RNA and DNA (Longley, Harkin & Johnston, 2003). 5-FU is also effective for the single or combined treatment of solid tumors and the prevention of liver metastasis in colon 26 adenocarcinoma cells in mice (Ishikawa et al., 1989; Tominaga et al., 1993). With the addition of 5-FU, the survival rates were significantly increased, and a marked improvement in the survival rates was produced by the combined treatment of 5-FU and H_2_.

At the lower dose, antioxidants may exhibit protective effect to the cells, including cancer cells, mainly through free radicals scavanger-like action. However, at the higher dose, antioxidants can lead cancer cell to apoptosis or enhancing apoptosis, probably due to excess free radicals (Nakashima-Kamimura et al., 2009; Oberley & Oberley, 1997; Simone et al., 2007a; Simone et al., 2007b). Similar actions were observed in the application of ascorbic acid. Treatment with both H_2_ waters (HHW and NHW) increased the expression of p-AMPK, AIF and Caspase 3 in colon 26 cells. Thus, H_2_ water resulted in cell apoptosis mediated by the AMPK pathway in colon 26 cells. Phosphorylation of AMPK appears to be involved in the apoptotic effect of H_2_ water in colon 26 cells, subsequently activating the caspase-dependent apoptosis pathways via caspase 3 and DNA degradation via AIF, as described previously (Jin & Reed, 2002). Although many signals are involved in apoptosis (Strasser, O’Connor & Dixit, 2000), H_2_-mediated cell apoptosis in colon 26 cancer cells is related to the AMPK pathway.

H_2_ enhanced the anticancer effect of 5-FU *in vivo*, which has not previously been demonstrated. Combined treatment of H_2_ water with 5-FU exhibited greater effectiveness against colon 26 cancer cells both *in vivo* and *in vitro*. This increased effectiveness might be due to the unique and non-overlapping mechanisms of action of the two agents. Hydrogen can also act as a scavenger of ROS, which may be produced secondarily by the tumor, leading to cell death via the caspase-independent pathway and lipid oxidation (Farmer & Mueller, 2013). Meanwhile, 5-FU functions solely through interfering with the process of DNA and RNA production in colon 26 (De Angelis et al., 2006; Longley, Harkin & Johnston, 2003). Therefore, H_2_ treatment is able to enhance the action of 5-FU to promote survival in colon 26-bearing mice and to increase the anticancer activities of 5-FU in cultured colon 26 cells. Furthermore, HHW resulted in anti-oxidative effects, higher cell apoptosis activities, and increased 5-FU efficacy more significantly than did NHW. These results are likely related to the higher H_2_ saturation in HHW (approximately 0.8 mM ) relative to NHW (approximately 0.1 mM ).

The limitation of present study is that we mainly focused on one particular cancer cell. We could not find the significant effect of H_2_ in HepG2 cancer cells. More colon and other cancer cells are required to investigate the detailed mechanisms and the efficacy of H_2_ in cancer.

In conclusion, the obtained data suggest that hydrogen water is able to produce cell apoptosis to enhance the anticancer activity of 5-FU in colon 26 cells both *in vivo* and *in vitro,* and these effects of hydrogen water are related to the hydrogen levels.

## Supplemental Information

10.7717/peerj.859/supp-1 Supplemental Information 1 Raw data
