# Supplementary figures and images for: Hydrogen–water enhances 5-fluorouracil-induced inhibition of colon cancer
Source: PeerJ. 2015 Apr 7;3:e859. doi: 10.7717/peerj.859 (PMC4393812; doi:10.7717/peerj.859)

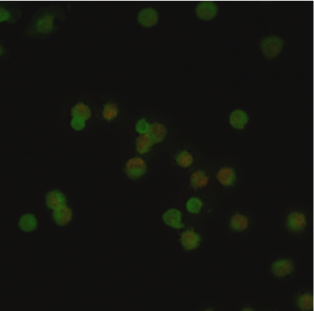

Supplement: Supplemental Information 1 [file peerj-03-859-s001.zip › Raw data/LIVE AND DEAD Raw data/5Fu COX 2.tif]

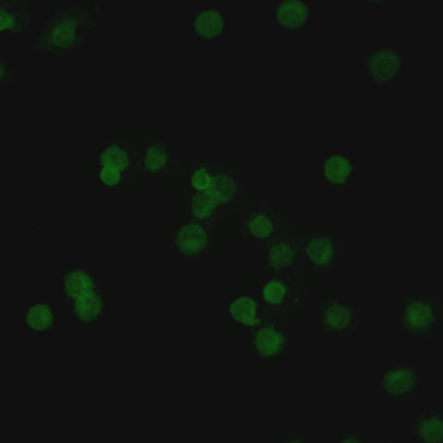

Supplement: Supplemental Information 1 [file peerj-03-859-s001.zip › Raw data/LIVE AND DEAD Raw data/5Fu Green 2.tif]

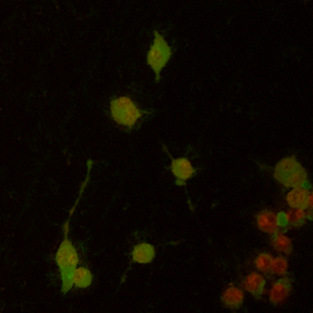

Supplement: Supplemental Information 1 [file peerj-03-859-s001.zip › Raw data/LIVE AND DEAD Raw data/5Fu H2 COX 5.tif]

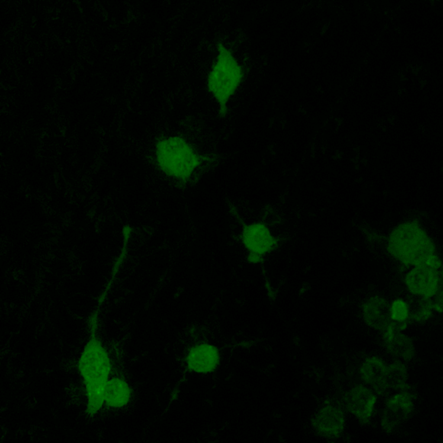

Supplement: Supplemental Information 1 [file peerj-03-859-s001.zip › Raw data/LIVE AND DEAD Raw data/5Fu H2 Green 5.tif]

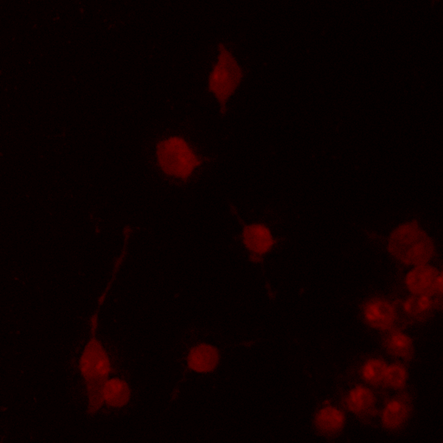

Supplement: Supplemental Information 1 [file peerj-03-859-s001.zip › Raw data/LIVE AND DEAD Raw data/5Fu H2 Red 5.tif]

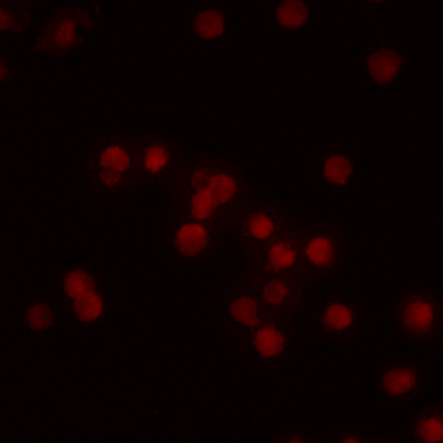

Supplement: Supplemental Information 1 [file peerj-03-859-s001.zip › Raw data/LIVE AND DEAD Raw data/5Fu Red 2.tif]

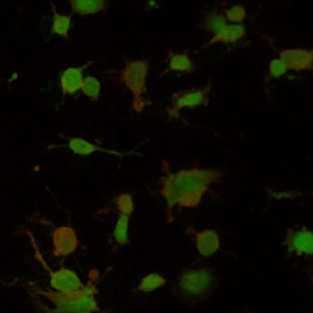

Supplement: Supplemental Information 1 [file peerj-03-859-s001.zip › Raw data/LIVE AND DEAD Raw data/5Fu VANA COX 6.tif]

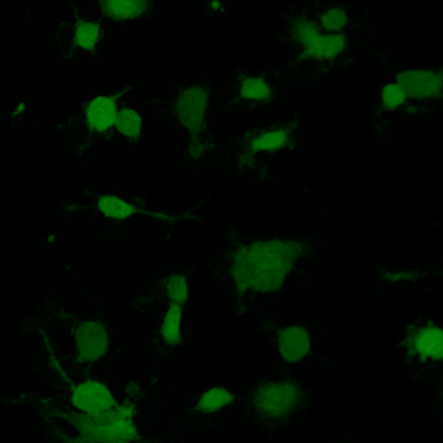

Supplement: Supplemental Information 1 [file peerj-03-859-s001.zip › Raw data/LIVE AND DEAD Raw data/5Fu VANA Green 6.tif]

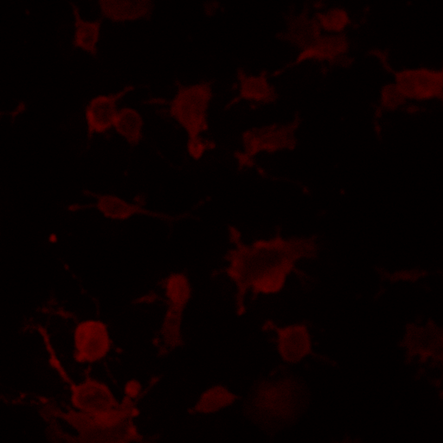

Supplement: Supplemental Information 1 [file peerj-03-859-s001.zip › Raw data/LIVE AND DEAD Raw data/5Fu VANA Red 6.tif]

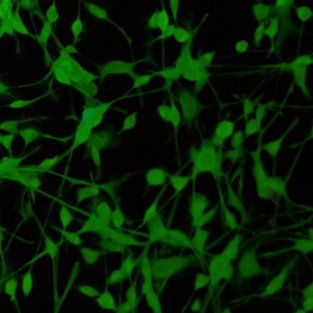

Supplement: Supplemental Information 1 [file peerj-03-859-s001.zip › Raw data/LIVE AND DEAD Raw data/Control COX 1.tif]

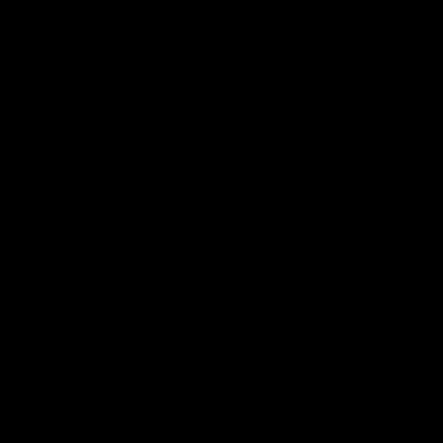

Supplement: Supplemental Information 1 [file peerj-03-859-s001.zip › Raw data/LIVE AND DEAD Raw data/Control Red 1.tif]

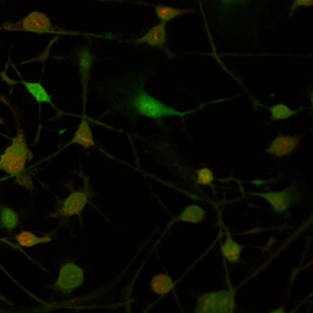

Supplement: Supplemental Information 1 [file peerj-03-859-s001.zip › Raw data/LIVE AND DEAD Raw data/H2 COX 3.tif]

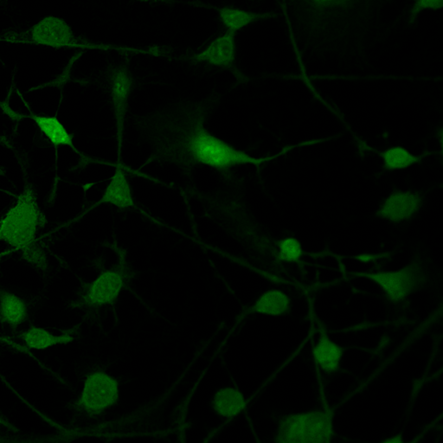

Supplement: Supplemental Information 1 [file peerj-03-859-s001.zip › Raw data/LIVE AND DEAD Raw data/H2 Green 3.tif]

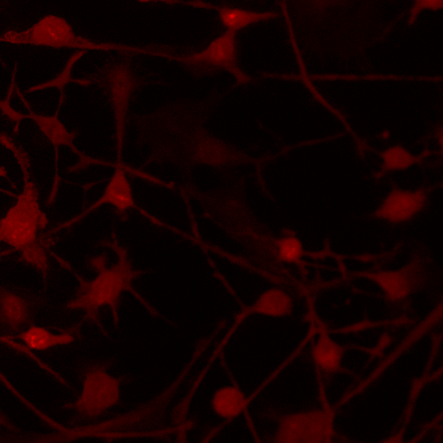

Supplement: Supplemental Information 1 [file peerj-03-859-s001.zip › Raw data/LIVE AND DEAD Raw data/H2 Red 3.tif]

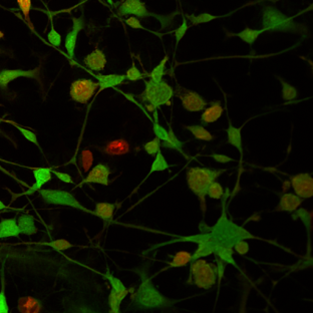

Supplement: Supplemental Information 1 [file peerj-03-859-s001.zip › Raw data/LIVE AND DEAD Raw data/VANA COX 4.tif]

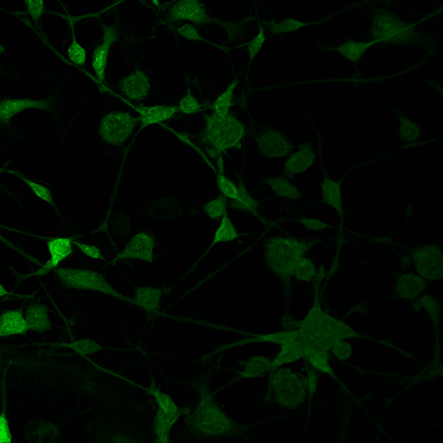

Supplement: Supplemental Information 1 [file peerj-03-859-s001.zip › Raw data/LIVE AND DEAD Raw data/VANA Green 4.tif]

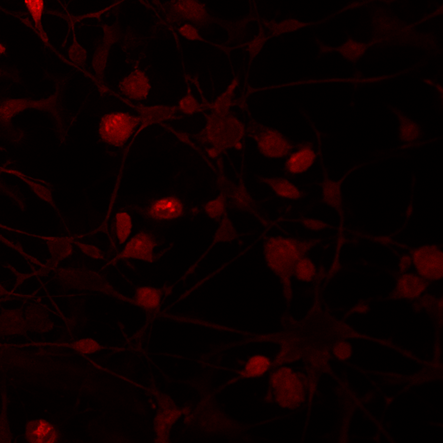

Supplement: Supplemental Information 1 [file peerj-03-859-s001.zip › Raw data/LIVE AND DEAD Raw data/VANA Red 4.tif]
